# Long non-coding RNA CASC2 regulates osteoblasts matrix mineralization

**DOI:** 10.3389/fbioe.2023.1155596

**Published:** 2023-07-04

**Authors:** Jaime Freitas, Sara Reis Moura, Mário Adolfo Barbosa, Susana G. Santos, Maria Inês Almeida

**Affiliations:** ^1^ i3S—Instituto de Investigação e Inovação em Saúde, Universidade do Porto, Porto, Portugal; ^2^ INEB—Instituto de Engenharia Biomédica, Universidade do Porto, Porto, Portugal; ^3^ ICBAS—Instituto de Ciências Biomédicas Abel Salazar, Universidade do Porto, Porto, Portugal

**Keywords:** long noncoding transcripts, mesenchymal stem/stromal cells, bone, cell differentiation, extracellular matrix

## Abstract

Long non-coding RNAs (lncRNAs) are master regulators of gene expression and have recently emerged as potential innovative therapeutic targets. The deregulation of lncRNA expression patterns has been associated with age-related and noncommunicable diseases in the bone tissue, including osteoporosis and tumors. However, the specific role of lncRNAs in physiological or pathological conditions in the bone tissue still needs to be further clarified, for their exploitation as therapeutic tools. In the present study, we evaluate the potential of the lncRNA CASC2 as a regulator of osteogenic differentiation and mineralization. Results show that CASC2 expression is decreased during osteogenic differentiation of human bone marrow-derived Mesenchymal Stem/Stromal cells (hMSCs). CASC2 knockdown, using small interfering RNA against CASC2 (siCASC2), increases the expression of the late osteogenic marker Bone Sialoprotein (BSP), but does not impact ALP staining level nor the expression of early osteogenic transcripts, including RUNX2 and OPG. Although siCASC2 does not impact hMSC proliferation nor apoptosis, it promotes the mineralization of hMSC cultured under osteogenic-inducing conditions, as shown by the increase of calcium deposits. Mass spectrometry-based proteomic analysis revealed that 89 proteins are regulated by CASC2 at late osteogenic stages, including proteins associated with bone diseases or anthropometric and musculoskeletal traits. Specifically, the Cartilage Oligomeric Matrix Protein (COMP) is highly enhanced by CASC2 knockdown at late stages of osteogenic differentiation, at both transcriptional and protein level. On the other hand, inhibition of COMP impairs osteoblasts mineralization as well as the expression of BSP. The results indicate that lncRNA CASC2 regulates late osteogenic differentiation and mineralization in hMSC via COMP and BSP. In conclusion, this study suggests that targeting lncRNA CASC2 could be a potential approach for modulating bone mineralization.

## 1 Introduction

Regeneration of bone tissue, following fracture or tumor resection, presents important clinical challenges, in particular for large defects or impaired healing (Wildemann et al., 2021). These can lead to non-union or delayed union fractures, which occur in 5%–10% of the cases. Understanding the genetic program controlling bone mechanisms is crucial for the development of alternative gene-based strategies to support the current gold-standard treatments for damaged bone, which includes the use of autologous bone grafts or synthetic bone substitutes (Freitas et al., 2019). Recently, it has been shown that long non-protein-coding RNAs (lncRNAs) can function as epigenetic, transcriptional and post-transcriptional cellular regulators. LncRNAs control multiple regulatory programs, including cell differentiation, human mesenchymal stem/stromal cells (hMSCs) lineage commitment, osteogenesis, osteoclastogenesis and bone remodeling (Silva et al., 2019). During bone regeneration, hMSCs undergo a highly regulated cascade of events and orchestrated regulation of gene expression that culminate into bone-forming cells. Although whole transcriptome profiling studies revealed that lncRNAs are involved in distinct stages of osteogenic differentiation, only a small fraction of the annotated lncRNAs has been examined for their biological function (Spizzo et al., 2012; Silva et al., 2019).

The lncRNA Cancer Susceptibility candidate 2 (CASC2) is located at chromosome 10q26 and was initially identified as a tumor suppressor gene in several types of tumors, including lung, gastric, colorectal, and bladder, among others (Yu et al., 2018). Gain- and loss-of-function studies revealed that CASC2 is involved in cell growth, apoptosis, migration and invasion mechanisms (Luo et al., 2018; Yu et al., 2018; Zhang et al., 2019). Besides being a potential therapeutic target, CASC2 has also been explored as a disease biomarker (Yan et al., 2018; Wang et al., 2022). Recently, the role of CASC2 in bone-related diseases started to emerge. In osteosarcoma, the most frequent primary bone tumor, CASC2 levels are reduced compared with noncancerous cells, and its expression negatively correlates with more advanced stages of this tumor type (Ba et al., 2018). It also suppresses osteosarcoma proliferation, colony formation and invasion in a process partially mediated by miR-181a and RASSF6 (Ba et al., 2018). CASC2 also inhibits osteosarcoma progression in a subcutaneous mouse xenograft model (Lu et al., 2018). In osteoarthritis, an inflammatory joint disease that can cause damage to the bone, CASC2 expression have been shown to be upregulated in plasma samples and to correlate with proinflammatory IL-17 levels (Huang et al., 2019). Also, CASC2 overexpression reduces the proliferation and induces apoptosis of chondrocytes (Huang et al., 2019). This process is, at least in part, mediated by miR-93-5p, which overexpression leads to the downregulation of CASC2 in chondrocytes (Sun et al., 2020). However, the role of CASC2 in bone-related non-malignant and non-inflammatory conditions has not yet been revealed.

This study aims to investigate, for the first time, the involvement of CASC2 in the osteogenic differentiation of hMSC. We demonstrate that CASC2 is implicated in the terminal maturation of osteoblasts, specifically in the mineralization stage, and identified novel CASC2-regulated extracellular proteins.

## 2 Materials and methods

### 2.1 Primary bone marrow-derived hMSCs

hMSC were isolated from the discarded bone marrow of patients that underwent total hip arthroplasty or anterior cruciate ligament injury surgery at Centro Hospitalar de São João (CHSJ, Porto, Portugal), less than 24 h after collection (n = 8). Gender, age, site of bone marrow collection and type of surgery for each donor is provided in Supplementary Table S1. Commercially available hMSC from 1 bone marrow donor was purchased from Lonza. The protocol was approved by CHSJ Ethics Committee for Health, and follows the declaration of Helsinki. All patients signed a written informed consent. Briefly, the bone marrow cells were isolated through a lymphoprep (Cosmo Bio USA lnc) gradient density centrifugation, as previously reported (Moura et al., 2020). The white ring layer was collected, washed with Iscove’s Modified Dulbecco’s Medium (IMDM, Gibco) and viable cells were counted using trypan blue (Sigma-Aldrish) and a Neubauer chamber. Finally, the cells were plated in low-glucose Dulbecco’s Modified Eagle’s Medium (DMEM, Corning), with 10% (v/v) MSC-qualified heat inactivated fetal bovine serum (FBS, Gibco) and 1% (v/v) of penicillin/streptomycin (P/S, Invitrogen). After 72 h incubation at 37°C and 5% (v/v) CO_2_, the media was renewed, and the non-adherent cells were removed. Adherent cells were allowed to grow, detached with tryspsin/EDTA (Life Technologies) and sub-cultured with a cell density of 2,000 cells/cm^2^. The minimal criteria for defining multipotent mesenchymal stromal cells by the International Society for Cellular Therapy (Dominici et al., 2006) were used to confirm hMSCs purity.

### 2.2 Osteogenic differentiation

To induce osteogenic differentiation, primary hMSCs were cultured in low-glucose DMEM 10% (v/v) FBS, supplemented with dexamethasone (10^−7^ M, Sigma-Aldrich), β-glycerophosphate (10^−2^ M, Sigma-Aldrich) and ascorbic acid (5 × 10^−5^ M, Sigma-Aldrich). As a control, cells were grown in parallel without osteogenic supplements (basal conditions). Cell culture media was changed twice per week.

### 2.3 ALP and alizarin staining

hMSCs were first fixed with 4% (w/v) paraformaldehyde (PFA, Sigma-Aldrich), for 20 min at RT. The alkaline phosphatase (ALP) staining was performed by incubating the cells, for 45 min at RT (protected from light) with a solution of Fast Violet (0.25 g/L, Sigma-Aldrich) with Naphtol AS-MX Phosphatase alkaline solution (Sigma-Aldrich) (Almeida et al., 2016). The Alizarin staining was performed by incubating the fixed cells for 10 min at RT with an Alizarin Red S solution (10 g/L, Sigma-Aldrich). The staining solutions were then discarded and cells were washed at least 3 times with distilled water. Cells were visualized under the Leica DMi1 Inverted Microscope (Leica Microsystems) or the SZX10 stereo microscope (Olympus Life Science). The ALP and Alizarin staining were quantified in 5 random fields of each well and expressed as the ratio between the stained area and the total area analysed, using MATLAB (Moura et al., 2020).

### 2.4 RNA isolation

hMSCs were collected at day 3, 7, 14, 21 and 28 of culture under osteogenic differentiation or control conditions and total RNA was isolated using TRIzol reagent (Invitrogen) according to the manufacturer’s instructions. RNA concentration and purity were evaluated in a NanoDrop 1000 (Thermo Scientific) and RNA integrity was evaluated by agarose gel electrophoresis.

### 2.5 Reverse transcription and real-time quantitative polymerase chain reaction (RT-qPCR)

Total RNA was first treated with the TURBO DNA-free kit (Invitrogen), following the manufacturer’s protocol. Complementary DNA (cDNA) was then synthesized using the SuperScript IV Reverse Transcriptase kit (Invitrogen) with random hexamers (Invitrogen). The qPCR was carried out in a CFX96 Touch Real-Time PCR Detection System (Bio-Rad) with the iQ SYBR Green Supermix (Bio-Rad) and the following conditions: 3 min at 95°C and 40 cycles of 30 s at 95°C, 30 s at 58°C and 30 s at 72°C. Actin beta (ACTB) was used as a reference gene. All runs were performed in duplicate and the relative expression levels were calculated using the quantification cycle (C_q_) method, according to MIQE guidelines (Bustin et al., 2009). Primer sequences are shown in Supplementary Table S2.

### 2.6 Transfection with small interfering RNAs against CASC2 and COMP

hMSC were seeded in 6 (0.12 × 10^6^ cell/well) or 24 (0.06 × 10^6^ cells/well)-well plates and allowed to adhere overnight. On the day after, cells were transfected with 10 nM of a Silencer Select Pre-designed siRNA (Thermo Fisher Scientific) targeting the lncRNA cancer susceptibility candidate 2 CASC2 (siRNA ID n259834, siCASC2), the cartilage oligomeric matrix protein COMP (siRNA ID s3367, siCOMP) or a non-targeting negative control siRNA (siRNA ID 4390843, siCTRL), using Lipofectamine 2000 (Thermo Fisher Scientific), according to the manufacturer’s instructions. After transfection, cells were cultured for 48 h or 7 days before harvesting for RNA isolation. In addition, to assess the impact on ALP staining, hMSC were cultured in osteogenic inducing conditions for 7 days post-transfection. hMSCs were also allowed to undergo 14 days of osteogenic differentiation, before transfection and allowed to differentiate until day 16 (for RNA isolation, 48 h post-transfection) and day 21 (for RNA isolation, alizarin staining, proteomic analysis and immunofluorescence, 7 days post-transfection).

### 2.7 Measurement of metabolic activity by resazurin assay

To assess the effect of lncRNA CASC2 knockdown on the metabolism/proliferation, hMSCs transfected with either siCTRL or siCASC2 were seeded in 96-well plates. Resazurin (0.01 mg/mL, blue dye, Sigma-Aldrich) was added to the cells (10% v/v in regular growth media), and incubated for 2 h at 37°C and protected from the light. The experiment was performed with 7 technical replicates for each condition and in 3 different time-points: 24, 48 and 72 h post-transfection. The media containing the reduced resazurin (resorufin, pink dye) was transferred to a dark wall 96-well plate and fluorescence levels measured at an excitation wavelength of 530 nm and an emission wavelength of 590 nm, using the spectrophotometer microplate reader Synergy MX (Biotek Synergy).

### 2.8 Measurement of apoptosis by flow cytometry

For the assessment of siRNA-induced apoptosis, hMSCs transfected with either siCTRL or siCASC2 were harvested 48 h post-transfection and labelled with the FITC Annexin V Apoptosis Detection kit (BD Biosciences), according to manufacturer’s instructions. Briefly, cells were washed with PBS, re-suspended in binding buffer with FITC-Annexin V and Propidium Iodide (PI) for 15 min at RT in the dark. The percentage of labelled cells was then measured within 1 h, in a FACS Canto II flow cytometer (BD Biosciences) with BD FACS Diva software. Results were analysed using FlowJo Software.

### 2.9 Proteomic analysis

hMSCs (from Lonza) were cultured in osteogenic differentiation conditions for 14 days before transfection with the siCASC2 or siCTRL. Transfected cells were then cultured under osteogenic differentiation conditions for additional 7 days (until day 21) before being harvested, washed with cold PBS and lysed in cold RIPA buffer supplemented with protease inhibitors and phosphatase inhibitors, for 30 min. Cell lysates were centrifuged at 20,000 *g*, for 10 min, at 4°C and the protein-containing supernatant was quantified using a DC protein assay kit (Bio-Rad). Protein identification and label-free quantitation was performed by nanoLC-MS/MS, as described (Moura et al., 2020). Raw data was processed using Proteome Discoverer 2.3.0.523 software (Thermo Scientific). Protein identification was performed with Sequest HT search engine against the *Homo sapiens* entries from the UniProt database (https://www.uniprot.org/). Samples were normalized against to the total peptide signal and the quantitative evaluation of the detected peptides was performed using pairwise comparisons. Data was corrected using the Benjamin Hochberg method. Bioinformatics and data analysis was performed for proteins expressed in the 3 independent experiments with fold-change (FC) differences ≥1.25 or ≤ −1.25, with a minimum of two unique peptides, when comparing the siCASC2 against the siCTRL condition. Gene Ontology (GO) annotations of the differentially expressed proteins were collected using the Protein ANalysis THrough Evolutionary Relationships (PANTHER) tool (Mi et al., 2021) and the gene-levels association study was performed with the data available data at the Musculoskeletal Knowledge Portal (Kiel et al., 2020).

### 2.10 Immunofluorescence

hMSCs transfected with siRNAs targeting either the lncRNA CASC2 or the transcript COMP, or the respective siRNA controls, were washed and fixed with 4% (w/v) PFA in PBS. Cells were permeabilized with 0.25% (v/v) Triton in PBS prior to blocking and overnight incubation at 4°C with the primary antibody rabbit anti-COMP/Cartilage oligomeric matrix protein (ab231977; abcam). The secondary antibody anti-rabbit Alexa 488 (Cell Signaling Technologies) was incubated for 1 h at RT. Coverslips were mounted in microscope slides with Fluoroshield with DAPI (Sigma) and images randomly acquired in a Zeiss Axio Imager Z1. FIJI software was used for image analysis and quantification.

COMP distribution in bone samples from the femurs of rats, after being fixed in 10% (v/v) buffered formalin, decalcified in EDTA–glycerol solution and processed for paraffin embedding, was analysed by immunofluorescence staining. Antigen retrieval of the paraffin sections was performed through incubation with citrate buffer (10 mmol/L; pH = 6), for 20 min, at 95°C. Sections were blocked using a buffer containing 10% (v/v) FBS, 1% (w/v) BSA and 0.02% (v/v) triton X-100; and incubated overnight, at 4°C, with primary antibody against COMP (ab231977; abcam), following incubation with secondary antibody anti-rabbit Alexa 647.

### 2.11 Statistical analysis

Statistical analysis was performed using GraphPad Prism version 7 (GraphPad Software, Inc.). All experiments were repeated at least three times. Normality tests (Shapiro-Wilk normality test and Kolmogorov-Smirnov normality test) were used to assess normal (Gaussian) distribution. When data followed normal distribution, paired *t*-test was used to compare 2 groups. For non-parametric data, Wilcoxon signed-rank test was used. The statistical test used is identified in each figure legend. Statistical significance was considered for *p* < 0.05 (*p < 0.05; **p < 0.01).

## 3 Results

### 3.1 The expression of the lncRNA CASC2 is downregulated during the osteogenic differentiation of hMSCs

**FIGURE 1:**
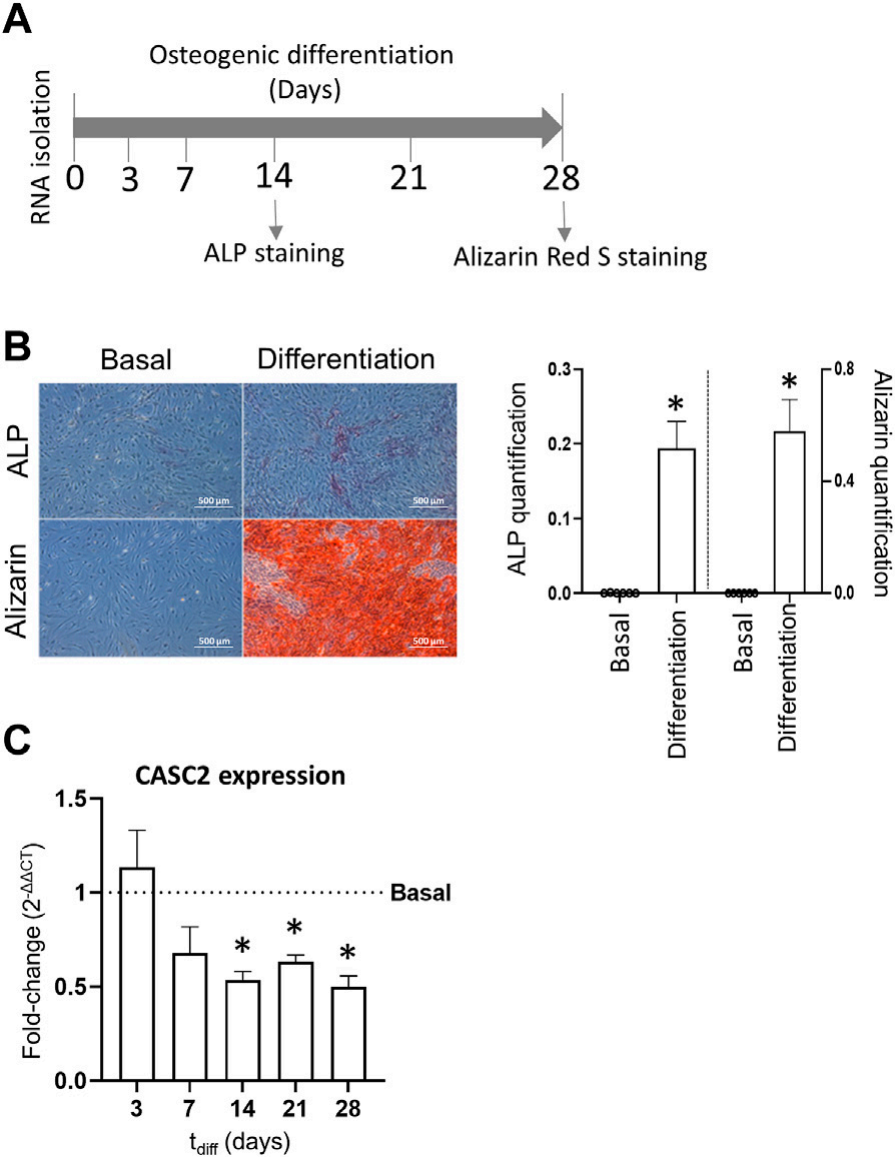
LncRNA CASC2 expression during osteogenic differentiation. **(A)**. Experimental timeline; **(B)**. ALP and Alizarin staining representative images and quantification in hMSCs cultured under basal and osteogenic differentiation conditions for 14 and 28 days, respectively (mean ± SEM, *n* = 6; *p < 0.05, Wilcoxon matched-pairs signed rank test); **(C)** CASC2 expression in hMSCs cultured under osteogenic conditions for 3, 7, 14, 21 and 28 days, compared with control conditions (mean ± SEM, *n* = 6; *p < 0.05, Wilcoxon signed rank test).

hMSCs were stimulated to differentiate into the osteogenic lineage for 28 days (Figure 1A). As expected, the early marker of osteoblast differentiation ALP is significantly increased at day 14, compared with hMSCs cultured in basal (non-osteogenic) conditions (Figure 1B). At later stages, stimulated cells significantly increased their matrix mineralization, measured through the alizarin staining of calcium deposits, which is associated with an efficient osteogenic differentiation at day 28 (Figure 1B). During the osteogenic differentiation, RNA samples were collected on days 3, 7, 14, 21 and 28 to assess the expression of CASC2. Results show that CASC2 transcript levels are consistently downregulated at late osteogenic differentiation stages (days 14, 21 and 28), but not at early stages (days 3 and 7), compared to the basal control cells (Figure 1C).

### 3.2 CASC2 downregulation increases matrix mineralization

**FIGURE 2:**
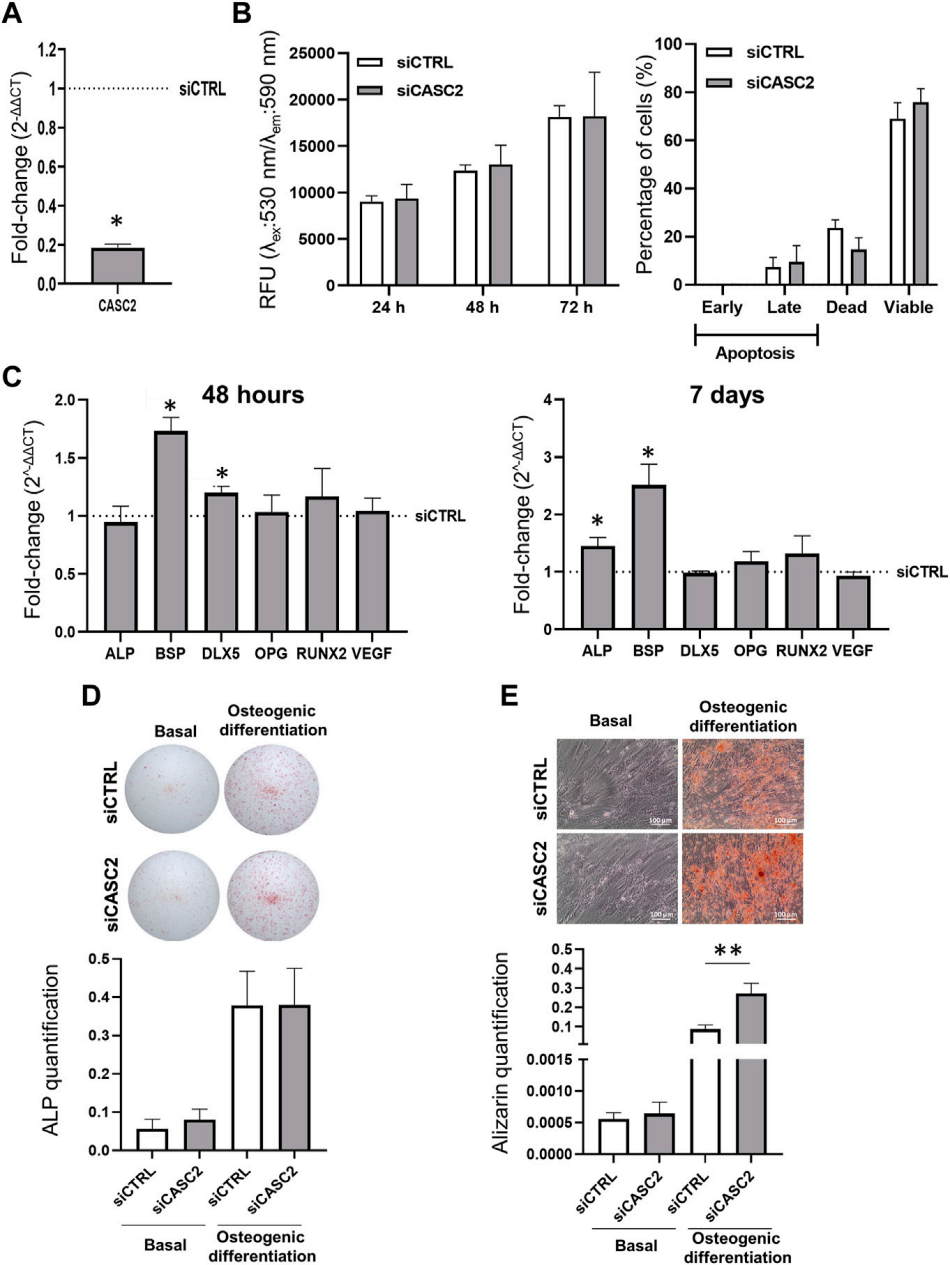
Effect of CASC2-knowckdown on proliferation, apoptosis and osteogenic differentiation. **(A)**. CASC2 expression in hMSCs 48 h post-transfection with a siRNA targeting the lncRNA CASC2 (siCASC2) (mean ± SEM, *n* = 6; *p < 0.05, Wilcoxon signed rank test); **(B)**. Resazurin-based cell viability analysis 24 h, 48 h, and 72 h after transfecting hMSCs with siCASC2 or siCTRL. Results are expressed as relative fluorescence units (RFU, mean ± SEM, *n* = 6). Apoptosis analysis 48 h after transfecting hMSCs with siCASC2 or siCTRL. Results show percent of cells in each cell stage (viable: Annexin V− PI−; early apoptotic: Annexin V + PI−; late apoptosis: Annexin V− PI+; dead cells: Annexin V + PI+, as determined by FACS analysis (mean ± SEM, *n* = 3); **(C)**. ALP, BSP, DLX5, OPG, RUNX2 and VEGF expression in hMSCs 48 h (left panel) and 7 days (right panel) post-transfection with a siRNA targeting the lncRNA CASC2 (siCASC2) in basal (non-osteogenic) conditions (mean ± SEM, *n* = 6; *p < 0.05, Wilcoxon signed rank test); **(D)**. ALP staining quantification in hMSCs transfected with siCASC2 or siCTRL and cultured for 7 days (D7) under basal and osteogenic differentiation conditions (mean ± SEM, *n* = 6). Representative images of ALP staining in siCASC2 or siCTRL transfected hMSCs cultured under basal and osteogenic differentiation conditions for 7 days are shown; **(E)**. hMSCs were cultured under basal and osteogenic differentiation conditions for 14 days before transfection with siCTRL and siCASC2. Alizarin staining quantification was performed at day 21 (mean ± SEM, *n* = 6; **p < 0.01, paired *t*-test). Representative images are shown below.

To determine the role of the lncRNA CASC2 in hMSC, we effectively silenced its expression, with an approximately 80% decrease 48 h post-transfection (Figure 2A), without inducing apoptosis or affecting cell metabolism (Figure 2B). Then, we measured the impact of CASC2 downregulation on the expression levels of osteogenic markers (Figure 2C). Interestingly, 48 h post-transfection with the siRNA targeting CASC2 we observe a significant 1.7-fold increase in the expression of bone sialoprotein II (BSP), an integral part of the mineralized matrix (Gordon et al., 2007; Farshdousti Hagh et al., 2015), and a 1.2-fold increase in the homeobox protein (DLX5), which is essential for osteoblast differentiation (Heo et al., 2017) (Figure 2C). Although BSP is mainly a marker of the onset of mineralization, we showed that BSP levels are upregulated following CASC2 reduction even at an early time point (48 h) in non-osteogenic culture conditions (Figure 2C). CASC2 expression is still significantly inhibited (approximately 85%) 7 days post-transfection with the siCASC2 (Supplementary Figure S1A). At this time point, BSP expression is also significantly increased with a 2.5-fold compared with siCTRL (Figure 2C). However, the increase in DLX5 expression is no longer observed 7 days post-transfection (Figure 2C). Interestingly, 7 days post-transfection with the siCASC2, we also observe a significant increase (1.5-fold) in the expression of the early marker of osteoblast differentiation ALP (Figure 2C), which suggests that CASC2 might be involved in the hMSCs progressive differentiation into osteoblasts. However, this does not translate into an increase in the ALP staining, either in basal or osteogenic inducing conditions (Figure 2D), which together with the sustained effect of siCASC2 in BSP expression (Figure 2C), a marker of late osteogenic differentiation, suggests that CASC2 has a greater impact on late stages of osteogenic differentiation rather than early stages. To validate this hypothesis, hMSCs were allowed to differentiate into osteoblasts for 14 days before the knockdown of CASC2 expression. The siCASC2 silencing efficiency 48 h post-transfection at day 14 is shown in Supplementary Figure S1B. Results show that siCASC2-transfected hMSCs in osteogenic differentiating conditions display a 3.17-fold increase in the mineralization capacity compared to siCTRL-transfected hMSCs (Figure 2E).

### 3.3 CASC2 downregulation affects the expression of proteins involved in bone mineralization

**FIGURE 3:**
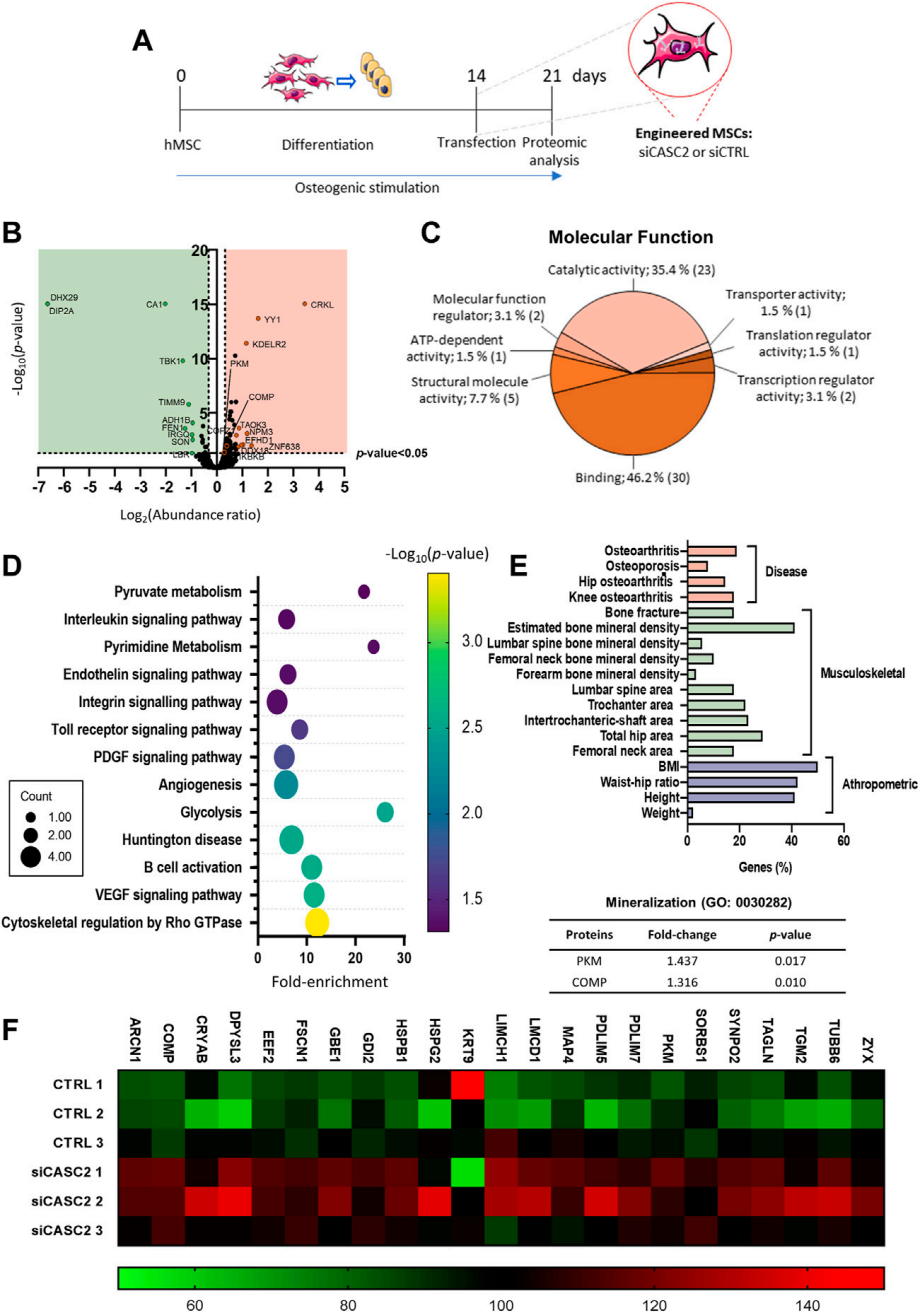
Proteomic analysis of the differently expressed proteins for CASC2-knockdown versus control. Commercial hMSCs were differentiated for 14 days in osteogenic inducing conditions before transfection with siCASC2 (n = 3). Proteins were collected 7 days after transfection. **(A)** Schematic representation of the experimental procedure; **(B)**. Volcano plot for the differently expressed proteins; **(C)**. GO analysis of the molecular functions for the differentially impacted proteins; **(D)**. Bubble plot of the enriched KEGG terms (*p* < 0.05; x-axis: fold-enrichment; color map: log10 (*p*-value) and circles size: number of proteins involved); **(E)**. Diseases and traits associated with the differently expressed proteins, following CASC2 silencing in osteoblasts; Fold-change and *p*-value for the proteins associated with the gene ontology term mineralization; **(F)**. Heatmap of the differently expressed proteins with unique peptides ≥15.

To identify novel CASC2-regulated molecules at late osteogenic differentiation stages, the total protein content of osteogenic differentiated hMSCs transfected with either siCASC2, or the respective negative control, at a late stage (Figure 3A), was isolated and analysed by mass spectrometry-based proteomics. Protein profiling identified a total of 3799 proteins (≥2 unique peptides), of which 89 proteins were differentially expressed between siCASC2-hMSC and scrambled control (*p* < 0.05), with 18 downregulated proteins (fold-change < −1.25) and 71 upregulated proteins (fold-change > −1.25) (Figure 3B).

Gene ontology (GO) analysis (downregulated and upregulated proteins) shows that the main molecular functions affected by the CASC2 silencing are the catalytic activity and binding (Figure 3C). The GO term catalytic activity (GO: 0003824) includes general child terms which are challenging to directly correlate with the observable increase in mineralization capacity, such as: acting on DNA, acting on RNA, acting on a protein, cyclase activity, demethylase activity, hydrolase activity, isomerase activity, ligase activity, lyase activity, oxidoreductase activity, transferase activity. Remarkably, the GO term binding (GO: 0005488) encompasses the child term extracellular matrix binding, which is described as a selective and non-covalent interaction with a component of the extracellular matrix. The analysis of the pathways associated to the differently expressed proteins by CASC2-knockdown revealed the involvement of several bone-related pathways, including cytoskeletal regulation by Rho GTPase and PDGF signaling (Figure 3D). Additionally, we used the Musculoskeletal Knowledge Portal (Kiel et al., 2020) to perform a gene-level association study for the same group of differentially expressed proteins. Interestingly, the studies show that a high percentage of the differentially expressed proteins are linked to several bone diseases (osteoarthritis and osteoporosis) or anthropometric and musculoskeletal traits (bone mineral density, femoral neck area and BMI) (Figure 3E). Since the main observable characteristic upon downregulation of the lncRNA CASC2 is an increase in the mineralization capacity of the osteogenic differentiated hMSCs, we investigated which of the differentially expressed proteins from the proteomic profiling were associated with bone mineralization (GO: 0030282). Of these, only the upregulated PKM (fold-change = 1.437; *p* = 0.017) and COMP (alias Thrombospondin-5; fold-change = 1.316; *p* = 0.010) had previously been implicated in bone mineralization (Figure 3E). Both are among the most differently expressed proteins with ≥15 unique peptides (Figure 3F).

### 3.4 CASC2 modulates hMSCs mineralization capacity through COMP

**FIGURE 4:**
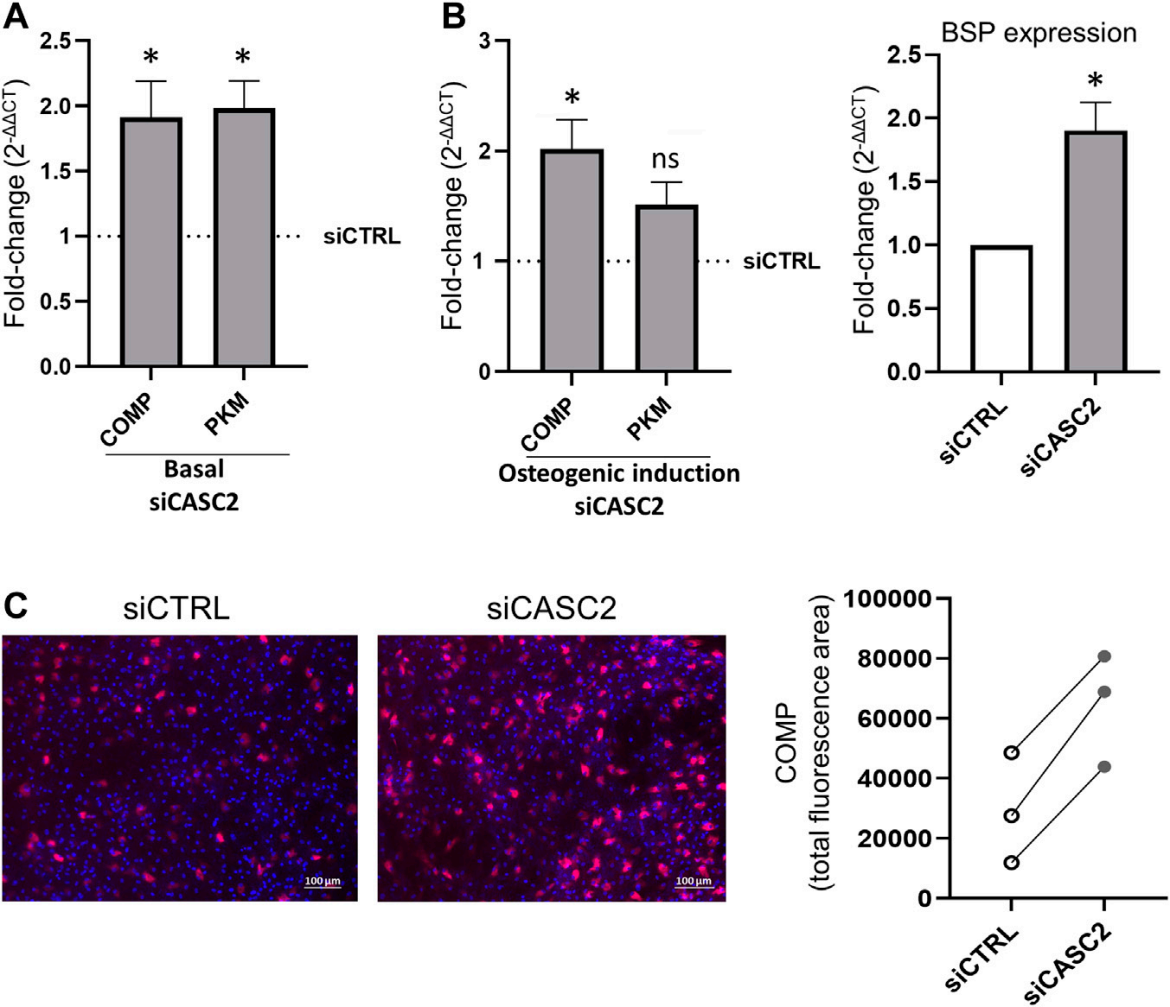
CASC2 downstream transcripts and proteins in hMSC. **(A)**. hMSCs cultured under basal conditions were transfected with siCTRL and siCASC2. COMP and PKM expression was measured 48 h after transfection (mean ± SEM, *n* = 6; *p < 0.05, Wilcoxon signed rank test); **(B)**. hMSCs were cultured under osteogenic differentiation conditions for 14 days before transfection with siCTRL and siCASC2. COMP, PKM and BSP expression was measured 48 h after transfection (mean ± SEM, *n* = 6; *p < 0.05, Wilcoxon signed rank test); **(C)**. Quantification of COMP-specific immunostaining 7 days post-transfection. hMSCs were cultured under osteogenic differentiation conditions for 14 days before transfection with siCTRL and siCASC2 (*n* = 3) Representative images are shown with COMP (red) and DAPI (blue) to visualize nuclei.

**FIGURE 5:**
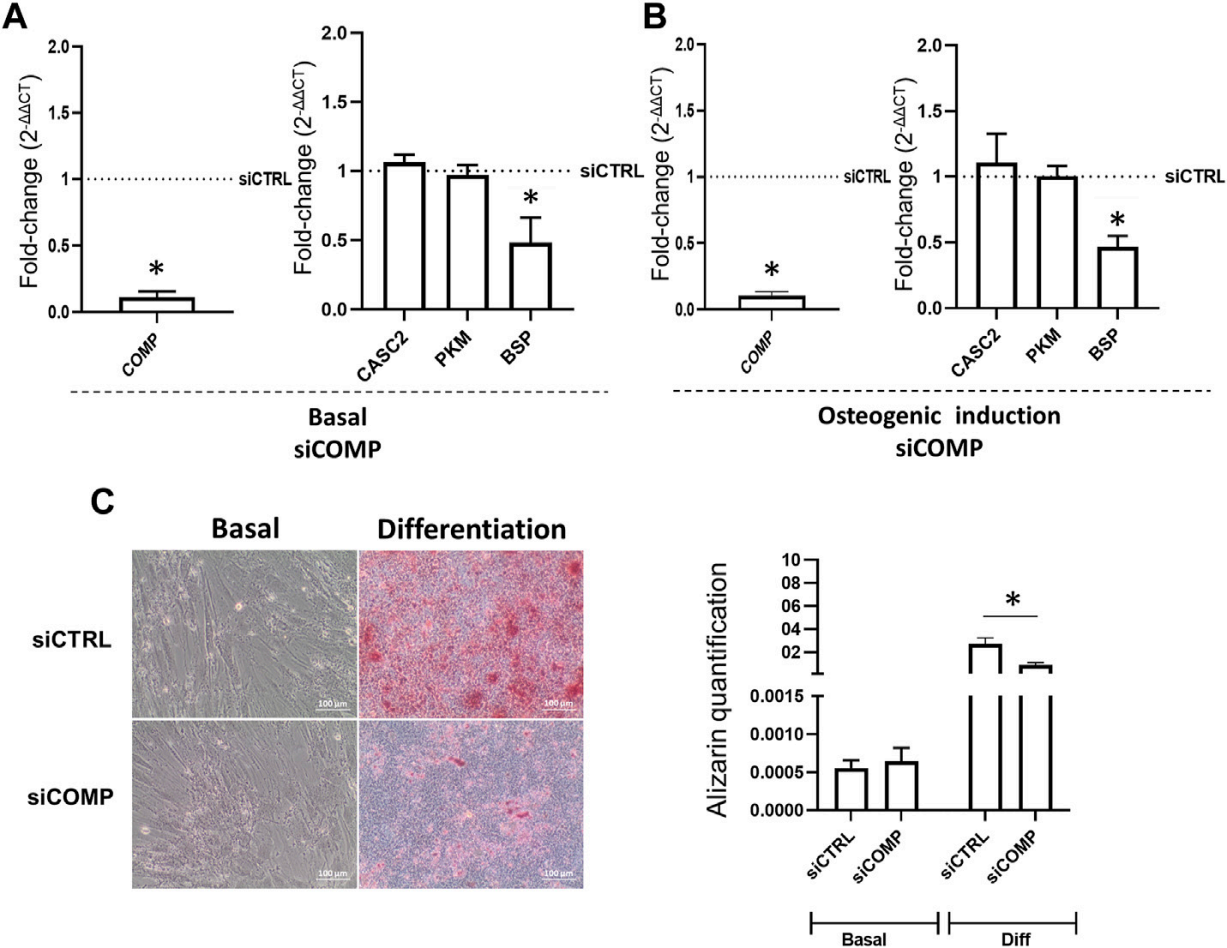
Reduction of COMP levels changes the expression of downstream transcripts and impacts mineralization. **(A)**. COMP, CASC2, PKM and BSP expression 48 h post-transfection with a siRNA targeting the gene encoding COMP (siCOMP), in hMSCs under basal conditions, (mean ± SEM, *n* = 6; *p < 0.05, Wilcoxon signed rank test); **(B)**. COMP, CASC2, PKM and BSP expression 48 h post-transfection with siCOMP, in hMSC previously cultured for 14 days under osteogenic differentiation conditions. (mean ± SEM, *n* = 6; *p < 0.05, Wilcoxon signed rank test); **(C)**. hMSCs were cultured under basal or osteogenic differentiation conditions for 14 days before transfection with siCTRL or siCOMP. Alizarin staining quantification was performed at day 21 (mean ± SEM, *n* = 6; **p < 0.01, paired *t*-test). Representative images are shown below.

Proteome profiling of siCASC2-transfected hMSCs has shown that reduction of CASC2 expression results in the upregulation of PKM and COMP, both involved in bone mineralization. The increased expression of PKM and COMP is observed 48 h post-transfection with siCASC2 even under basal conditions (Figure 4A). At late time points of osteogenic differentiation (14 days), CASC2 silencing also leads to increased expression of COMP and PKM, with fold-change increases of 2.02 and 1.52, respectively, although this increase is only statistically significant for COMP (Figure 4B). Additionally, the marker BSP was found upregulated in siCASC2-transfected hMSC at late differentiation stages (Figure 4B). The result for COMP was further validated by immunofluorescence (Figure 4C). Next, we used a pre-designed siRNA targeting the gene encoding COMP (siCOMP) to knockdown its expression. Under basal conditions, COMP expression levels were decreased by approximately 90% compared to the control, 48 h post-transfection (Figure 5A), which also resulted in a decrease in the expression of BSP, without affecting CASC2 or PKM expression (Figure 5A). Similarly, silencing the expression of COMP at day 14 of osteogenic differentiation also results in an approximately 90% decrease in its expression (48 h post-transfection, Figure 5B), and once again only affects the expression of BSP (Figure 5B). The decrease in COMP at the protein level, after siCOMP transfection, has also been validated (Supplementary Figure S2). As expected, transfection with siCOMP of hMSCs-differentiated osteoblasts at day 14 resulted in a significantly lower mineralization capacity compared to the control (Figure 5C). Considering that the results show that COMP regulates mineralization, we next performed COMP-specific immunostaining in healthy rat femurs to determine COMP location and distribution within the bone tissue. Differences regarding quantity and location of COMP were detected, with a lower but detectable accumulation of COMP in the subchondral bone compared with cartilage tissue (Supplementary Figure S3).

## 4 Discussion

LncRNAs have been increasingly recognized as regulators of bone-related processes (e.g., osteogenic differentiation, osteoclastogenesis, bone resorption) and their deregulation may lead to bone diseases (e.g., osteoporosis) (Silva et al., 2019). In this study, we identified CASC2 as a novel negative regulator of osteoblasts mineralization. Herein, CASC2 has a minor impact on the early stages of osteogenic differentiation in hMSC. We show that silencing of CASC2 does not impact RUNX2 expression nor ALP staining. Also, there is a consistent reduction of CASC2 levels in the late time points of osteogenic differentiation in hMSC, but not at the early stages. The impact of this gene was best revealed when CASC2 expression was silenced on day 14 of osteogenic differentiation. The results presented support CASC2 as a mineralization-specific lncRNA in hMSC-derived osteoblasts, which mediates the late osteogenic differentiation stages, but not the early or hMSC commitment phase.

Both hypo- and hyper-mineralization are detrimental for the skeletal function and may lead to disease (Bourne et al., 2021). Thus, controlled regulation of this process can prevent bone-related diseases. As such, CASC2 can be a potential target to modulate mineralization. Additionally, considering that chondrocyte hypertrophy is an essential contributor to endochondral ossification, and that mineralization is critical during this process (Sun and Beier, 2014; Bourne et al., 2021), potential consequences can be expected when modulating CASC2 expression (Sun and Beier, 2014).

Furthermore, this study identified two main extracellular proteins regulated by CASC2, specifically BSP and COMP. BSP is a multifunctional noncollagenous glycoprotein abundantly expressed in mineralized tissues and predominantly associated with the bone ECM (Gordon et al., 2007). BSP plays an essential role in osteoblast-mediated biomineralization (Gorski, 2011). On the other hand, the role of COMP in bone has been less characterized. COMP is a non-collagenous glycoprotein and a member of the pentameric subgroup of the thrombospondin gene family, which is present in the ECM of different tissues and cells, including cartilage, fibroblasts, vascular smooth cells, among others (Posey et al., 2018). This protein interacts with other ECM components, growth factors, and cell surface receptors (Acharya et al., 2014). The role of COMP in articular cartilage has been widely studied, where it is abundantly expressed (Acharya et al., 2014). COMP levels are reduced in cartilage diseases such as OA, and mutations in COMP lead to skeletal disorders such as pseudoachondroplasia and multiple epiphyseal dysplasia (Song et al., 2003). However, COMP is also expressed in embryonic and adult osteoblasts (Di Cesare et al., 2000), and has been shown to play a role in osteogenesis (Coustry et al., 2018). Specifically, it can bind to BMP-2 enhancing the activation of the downstream SMAD signaling pathway and can increase BMP2-induced bone formation (Ishida et al., 2013). Supplementation of COMP to *in vitro* C2C12 cultures caused an increase in OPN (Ishida et al., 2013), a marker of osteoblasts maturation (Si et al., 2020). Our study shows that the reduction of COMP impairs mineralization of hMSC-derived osteoblasts in osteogenic-inducing conditions. Also, we reveal herein, for the first time, that COMP is a downstream target of the lncRNA CASC2.

## 5 Conclusions

In conclusion, our study identified a role for CASC2 in osteoblasts mineralization. CASC2 expression profile in hMSCs shows that this lncRNA is downregulated at late stages of osteogenic differentiation. Reduction of CASC2 levels leads to an increase in the alizarin staining, while proteomic profiling analysis shows it can influence targets involved in bone-related diseases and pathways. COMP was identified as a novel CASC2 downstream target in MSC-derived osteoblasts. We show that the inhibition of COMP reduces mineral deposition. Globally, our study supports the modulation of CASC2 levels as a molecular therapeutic tool to control bone mineralization.

## Data Availability

The datasets used during the current study have been deposited to the ProteomeXchange Consortium via the PRIDE partner repository with the dataset identifier PXD040463.

## Supplementary material

SUPPLEMENTARY FIGURE S1 CASC2 levels post-transfection with siCASC2. **(A)** CASC2 expression in hMSCs 7 days post-transfection with a siRNA targeting the lncRNA CASC2 (mean±SEM, *n*=6). **(B)** hMSCs were cultured under osteogenic differentiation conditions for 14 days before transfection with siCTRL and siCASC2. CASC2 expression was measured 48 h after transfection (mean±SEM, *n*=6).

SUPPLEMENTARY FIGURE S2 COMP immunostaining. hMSC were cultured under osteogenic differentiation conditions for 14 days before transfection with siCOMP. Protein levels were quantified 7 days post-transfection (*n*=3). Representative images are shown with COMP (magenta) and DAPI (blue) to visualize nuclei.

SUPPLEMENTARY FIGURE S3 COMP-specific immunostaining in healthy rat femur. Representative images are shown with COMP (magenta) and DAPI (blue) to visualize nuclei.

SUPPLEMENTARY TABLE S1 Human MSCs donor profile.

SUPPLEMENTARY TABLE S2 Primer sequences.
