# Supplementary figures and images for: Long non-coding RNA CASC2 regulates osteoblasts matrix mineralization
Source: Front Bioeng Biotechnol. 2023 Jul 4;11:1155596. doi: 10.3389/fbioe.2023.1155596 (PMC10353537; doi:10.3389/fbioe.2023.1155596)

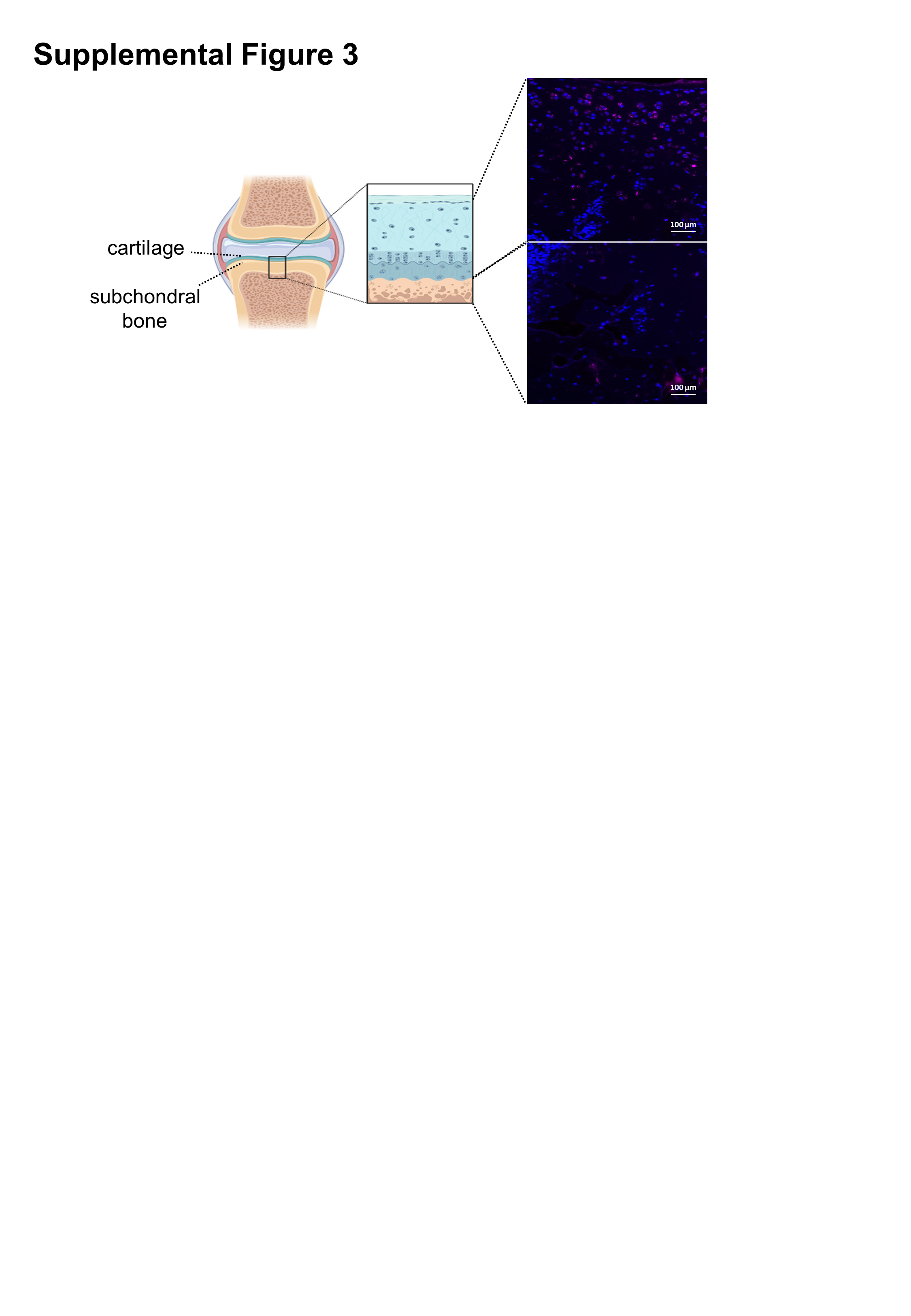

Supplement: Supplementary file 1 [file Image3.tif]

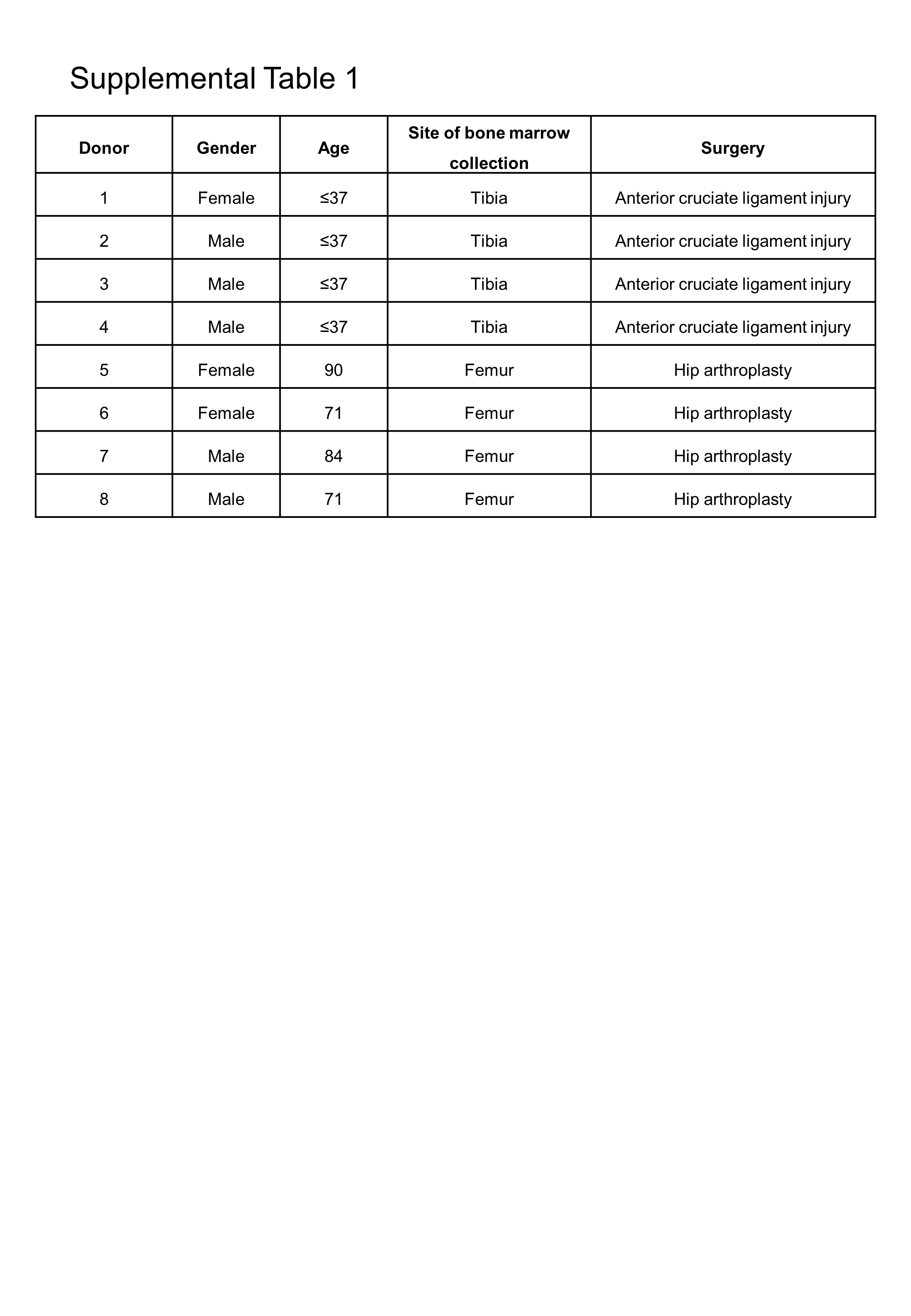

Supplement: Supplementary file 2 [file Image4.TIF]

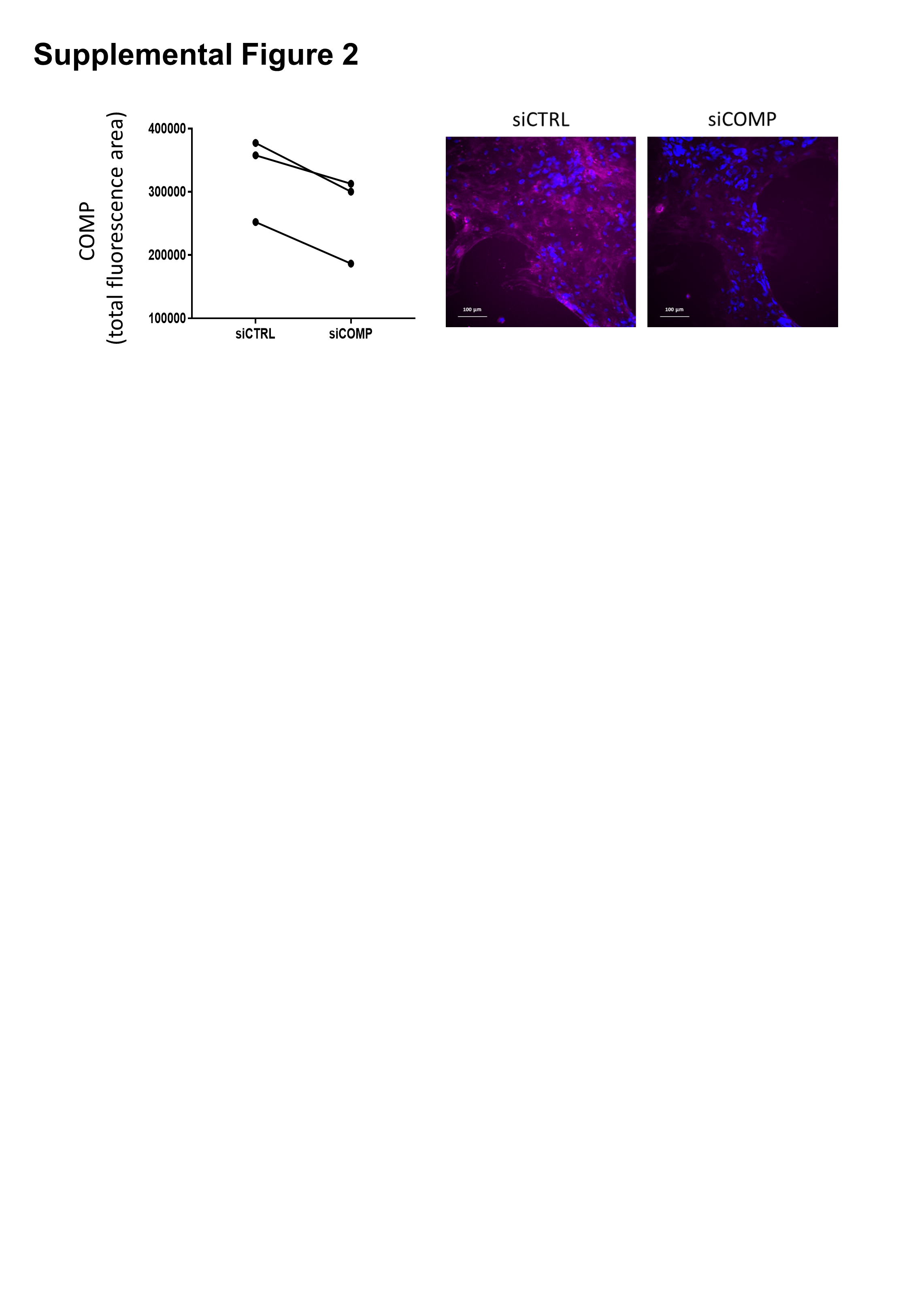

Supplement: Supplementary file 3 [file Image2.tif]

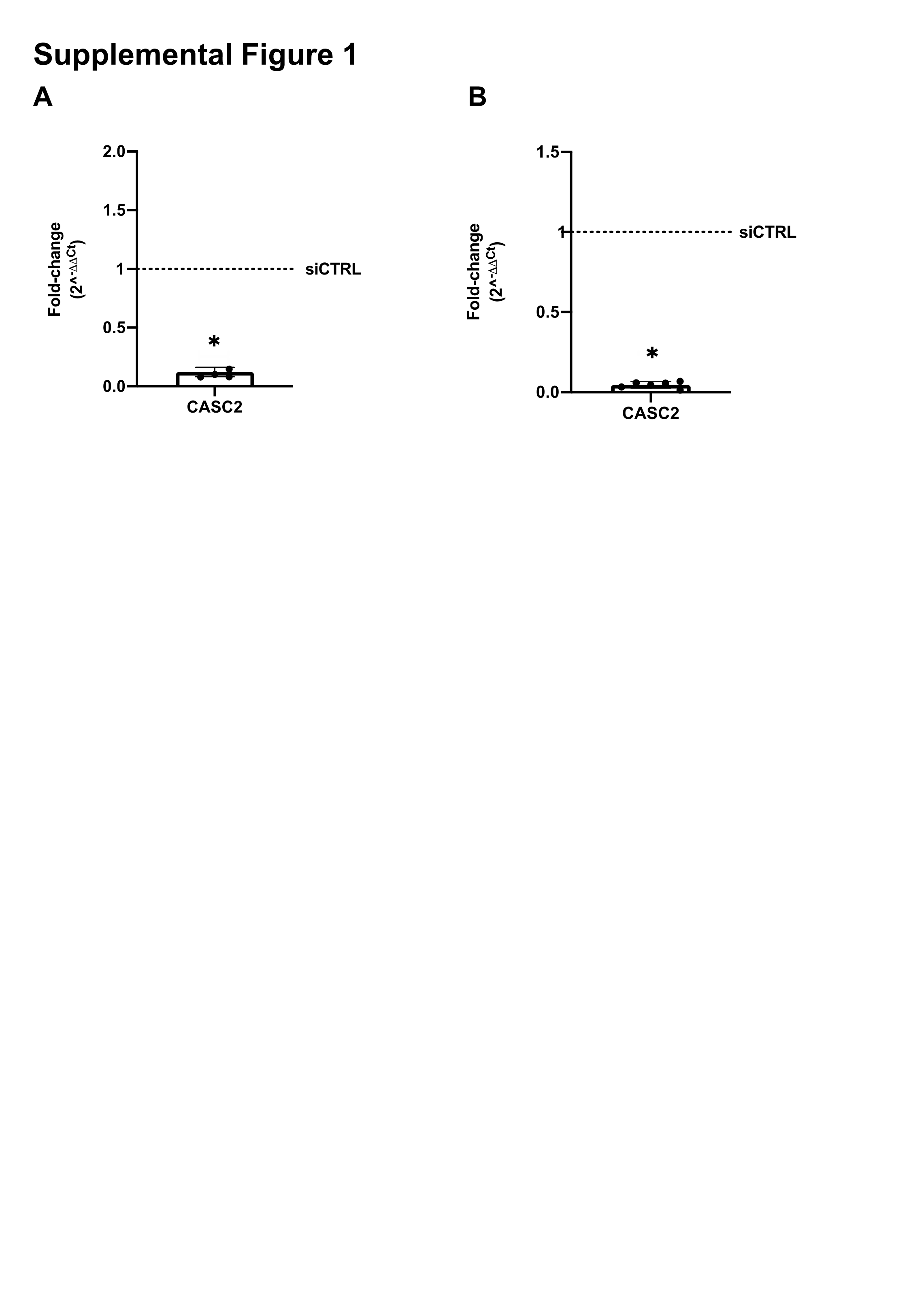

Supplement: Supplementary file 4 [file Image1.tif]

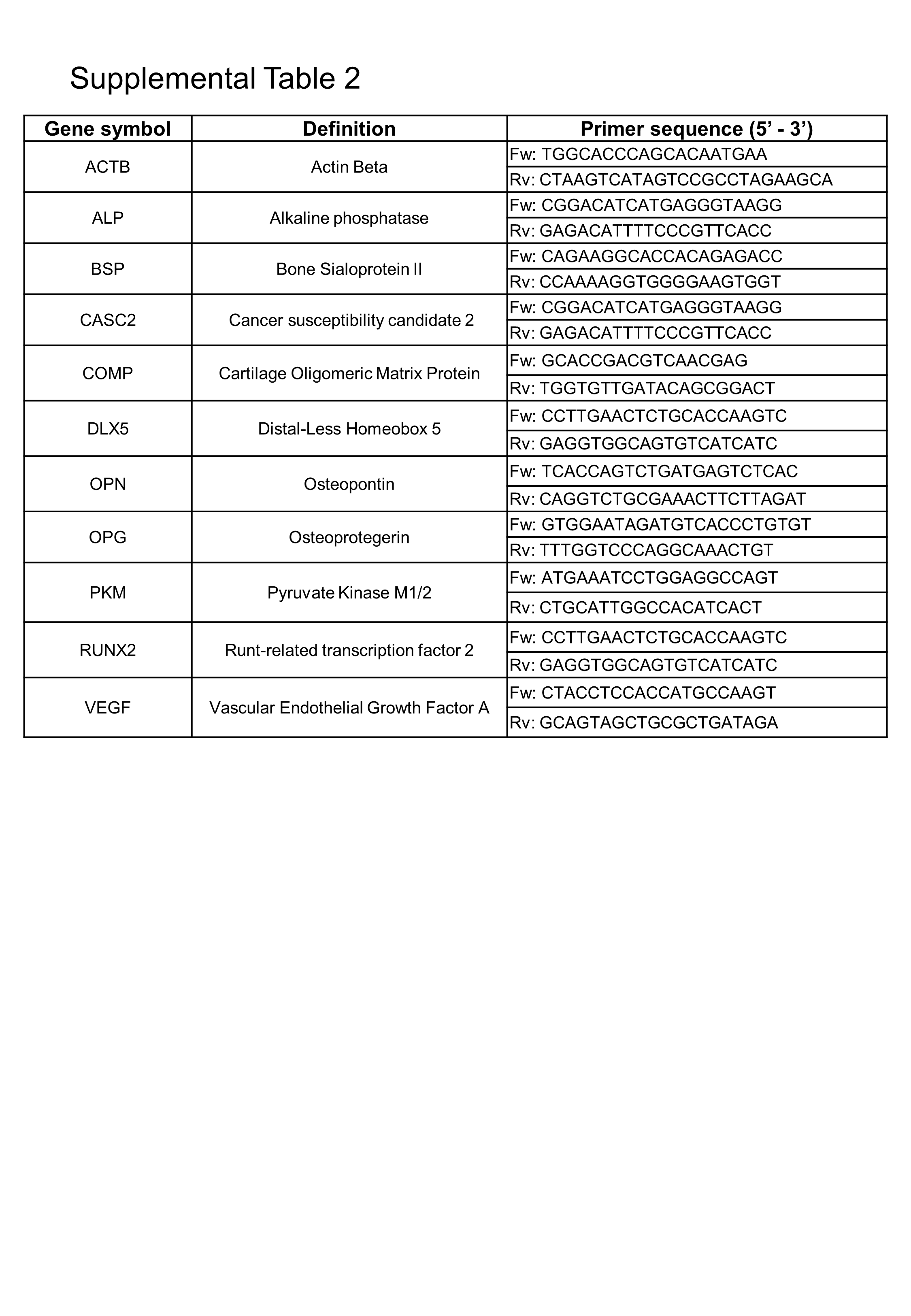

Supplement: Supplementary file 5 [file Image5.TIF]
